# Supplementary material for: Development and Validation of the Attitudes towards Social Robots Scale
Source: Healthcare (Basel). 2024 Jan 23;12(3):286. doi: 10.3390/healthcare12030286 (PMC10855967; doi:10.3390/healthcare12030286)
Supplement: Supplementary file 1 [file healthcare-12-00286-s001.zip › healthcare-2776969-supplementary.pdf]

ASRS Item list

Ich würde einen intelligenten Roboter als Freund und Helfer an meiner Seite schätzen. (ASRS 01)

Ein Pflegeroboter, der sich um mich kümmert und sich im Alter um mich kümmert, wäre schön (ASRS 02)

Ein menschenähnlicher Roboter könnte sich um meine Eltern kümmern, wenn sie alt werden (ASRS 03)

Ich würde einen Roboter auf meine Kinder aufpassen lassen. (ASRS 04)

Ich glaube, dass Roboter eines Tages ein Bewusstsein entwickeln könnten, das mit dem des Menschen vergleichbar ist. (ASRS 05)

Wenn Roboter ein Bewusstsein entwickeln, dann sollte es für sie Rechte geben, die den menschlichen Rechten vergleichbar sind (ASRS 06)

Wenn es Roboter gäbe, die von Menschen nicht zu unterscheiden wären, würde ich in Erwägung ziehen, einen Roboter zu heiraten (ASRS 07)

Ich könnte mir auch sexuellen Kontakt mit Robotern vorstellen. (ASRS 08)

Auch wenn Roboter ein Bewusstsein entwickeln sollten, bleiben es Maschinen (ASRS 09)
